# Dexmedetomidine potently and reversibly regulates stress-mediated behaviors

**DOI:** 10.3389/fphar.2025.1589075

**Published:** 2025-07-02

**Authors:** Frank D. Yocca, Dinesh K. Dhull, Subhendu Seth, Manisha Chugh, Sameer Sharma, Krishnan Nandabalan, Friso R. Postma, Michael De Vivo

**Affiliations:** ^1^ BioXcel Therapeutics Inc., New Haven, CT, United States; ^2^ E. Z. BioXcel Solutions Pvt. Ltd., Gurugram, Haryana, India; ^3^ Invea Therapeutics Inc., Guilford, CT, United States

**Keywords:** dexmedetomidine, stress, Alpha2-Adrenoceptor, locus coeruleus, anxiety, agitation, noradrenergic, aggression

## Abstract

**Aims:**

Demonstrate that dexmedetomidine is potentially useful for treatment of psychiatric symptoms caused by acute or chronic stress by using relevant rodent models and doses predicted to achieve sufficient brain levels to activate alpha2-adrenoceptors.

**Methods:**

*In vitro* characterization was performed using labeled GTPγS binding and β-arrestin recruitment. Free brain levels were measured by using microdialysis probes in rats. *In vivo* behavioral characterization of dexmedetomidine was performed both an acute stress model (forced swim test or despair test) and a repeat stress model with repeat dosing (the open space swim test). Dexmedetomidine was assessed using translational models: the CCK-4 induced panic test in Wistar rats and latency to rapid eye movement sleep (REM sleep). Rotarod assessments (Swiss mice) and a cued memory consolidation model (Wistar rats) were used to ensure that effective doses had no overt effects on motor coordination or memory consolidation.

**Results:**

Dexmedetomidine is both more potent (<10 nM EC50) and has higher intrinsic activity than other α2-AR agonists (clonidine, lofexidine or guanfacine). Estimates of free brain levels at efficacious doses are sufficient to activate all 3 α2-ARs. Dexmedetomidine was effective in decreasing immobility (increasing escape behaviors) in the forced swim test after a single acute stress and in the open space swim test after repeat dosing. Effects of dexmedetomidine were completely reversible and no withdrawal effects were observed. Doses used in the open space swim test had no overt effect on the rotarod indicating no impairment of motor coordination. Dexmedetomidine was highly effective in reversing a CCK-4 induced mediated deficit in the elevated plus maze, a translatable model for anxiety and panic. Dexmedetomidine also increased latency to REM sleep and shortened latency to slow wave sleep, positive attributes for a neuropsychiatric drug.

**Conclusion:**

Dexmedetomidine may be a suitable drug for a chronic dosing for a wide range of stress-mediated symptoms, not limited to the acute treatment of agitation. Brain levels are highly predictable based on plasma exposures and are consistent with the known affinity for α2-ARs.

## 1 Introduction

Pharmacological treatment of agitation in patients with psychiatric disorders is largely limited to benzodiazepines and antipsychotic drugs which modulate GABAergic and dopaminergic signaling, respectively. Recently BXCL501 has been approved by the United States Food and Drug Administration (FDA) for the acute treatment of episodes of agitation in patients diagnosed with bipolar disorder or schizophrenia (Citrome et al., 2022; Preskorn et al., 2022; Zagorski, 2022). The active ingredient in BXCL501 is dexmedetomidine, an alpha2-adrenoceptor (α_2_-AR) agonist, the same class of drug as clonidine, lofexidine and guanfacine. Dexmedetomidine is a novel anti-agitation drug in that it reduces noradrenergic signaling. Dexmedetomidine accomplishes this by activating α_2_-ARs expressed on locus coeruleus (LC) neurons, the main norepinephrine-containing neurons in the CNS. These α_2_-ARs act as autoreceptors and reduce the release of norepinephrine from LC neurons. Multiple lines of evidence support heightened LC activity and subsequent norepinephrine signaling as a likely mechanism for stress-mediated psychiatric symptoms such as anxiety and agitation. In this study, we characterize the preclinical *in vitro* and *in vivo* profile of dexmedetomidine to better inform the clinical use of the drug.

There are 3 genes in the α_2_-AR family: ADRA2A, ADRA2B and ADRA2C, encoding the G-protein coupled receptors (GPCRs) α_2A_-AR, α_2B_-AR and α_2C_-AR. GPCRs generate signals by at least 2 pathways: increased binding of GTP to of G-proteins or by recruitment of β-arrestin to the plasma membrane (Fink et al., 2022). In this study, the potency and intrinsic activity of dexmedetomidine at each of the 3 α_2_-ARs was measured using G-protein activation or β-arrestin recruitment. Free brain levels after dosing dexmedetomidine were measured using microdialysis probes and compared to the *in vitro* potency of dexmedetomidine at the α_2_-ARs to ensure that the effects measured in the rodent behavior studies were likely mediated by α_2_-ARs and not off-target effects.

Dexmedetomidine was assessed both after an acute stress (the forced swim test) and after repeated stress (the open space swimming test), both known to be dependent on noradrenergic signaling. Doses used in these efficacy studies were tested in models that assess motor coordination and memory consolidation. Finally, to assess the relevance of these stress models to psychiatric symptoms, we evaluated dexmedetomidine in 2 models with high translational validity, latency to rapid eye movement (REM) sleep and the CCK4-induced panic anxiety test. The objective of this study was to demonstrate that dexmedetomidine may be broadly useful for the chronic treatment of stress-related psychiatric disorders and not limited to acute treatment of agitation, its approved use.

## 2 Materials and methods

### 2.1 Materials

*In vitro* assays: Dexmedetomidine HCl (SML0956) and other test compounds: lofexidine HCl (SML1019), guanfacine HCl (G1043), and clonidine HCl (C7897) were purchased from Sigma-Aldrich (MO, United States). *In vivo* experiments: Dexmedetomidine HCl was purchased from either MedChemExpress, NJ, United States (HY-17034A) or Sigma-Aldrich, Bangalore, India (SML0956).

Drugs used as positive controls, and other standard reagents, were supplied by respective CROs.

### 2.2 [^35^S]GTPγS binding assay

#### 2.2.1 Site

GTPγS bidding assay was performed by Eurofins Panlabs Discovery Services Taiwan, Ltd. (New Taipei City, Taiwan).

#### 2.2.2 Procedure

Cells were transfected with constructs encoding either human α_2A_ (Chem-1, rat hematopoietic cell line), or α_2B_ or α_2C_ (Cho-K1, Chinese hamster ovary cell line) adrenergic receptors. Each test compound was prepared in DMSO at 10 mM and diluted to indicated concentrations in the incubation buffer (20 mM HEPES, 100 mM NaCl, 10 mM MgCl_2_, 1 mM DTT, 1 mM EDTA, pH 7.4.

The assay was performed as described in detail by Audinot et al. (2002). After recombinant receptors expression, the cells were harvested, cell membranes were collected and incubated with [^35^S]GTPγS. The amount of [^35^S]GTPγS, incorporated after incubation with the agonists (dexmedetomidine, clonidine, guanfacine, lofexidine, or control compound UK-14304) was determined. Binding data were generated as duplicates. Data were analyzed with GraphPad Prism (GraphPad Prism^®^ Software, San Diego, CA) using a 3-parameter logistic equation to yield EC_50_ values (concentration of compound that gives 50% of stimulation of [^35^S]GTPγS binding). Intrinsic activity was normalized using the maximal response achieved by the positive control, UK-14304, which was tested in each experiment. Three parameter logistic equations were fitted to the data to obtain EC_50_ and E_max_ values.

### 2.3 β-Arrestin chemiluminescent assay

Relative potency and intrinsic activity of alpha-2 adrenergic agonists were determined by measuring agonist-induced increases in recruitment of beta-arrestin using the PathHunter system (Wang et al., 2004). PathHunter cell lines were expanded from freezer stocks according to standard procedures. Cells were seeded in a total volume of 20 μL into white walled, 384-well microplates and incubated at 37°C for the appropriate time prior to testing. Intermediate dilution of sample stocks was performed to generate 5X sample in assay buffer. 5 μL of 5X sample was added to cells and incubated at 37°C or room temperature for 90–180 min. Vehicle concentration was 1%. Assay signal was generated through a single addition of 12.5 or 15 μL (50% v/v) of PathHunter Detection reagent cocktail, followed by a 1-h incubation at room temperature. Microplates were read following signal generation with a PerkinElmer Envision™ instrument for chemiluminescent signal detection.

### 2.4 *In Vivo* microdialysis

#### 2.4.1 Site

Studies were conducted by Charles River Laboratories (South San Francisco, CA). Experiments were conducted in accordance with protocols approved by the Institutional Animal Care and Use Committee of Charles River Laboratories, SSF.

#### 2.4.2 Animals

Male Sprague Dawley (SD) rats, 200–250 g, purchased from Charles River Laboratories (South San Francisco, CA) were used for microdialyses studies. Upon arrival, rats were group-housed in polycarbonate cages (2-3 rats per cage) and acclimated for at least 7 days prior to commencing the studies. Animals were housed in a 12-h light/dark cycle with room temperature maintained at 22 ± 2^o^C and approximately 50% humidity and received food and water *ad libitum*. Rats were tracked via unique identifying numbers.

#### 2.4.3 Procedure

Ten male SD rats with a jugular vein (JV) cannula were used in this study. Rats were anesthetized using isoflurane (2%, 800 mL/min O2). Bupivacaine was used for local anesthesia and carprofen for peri-/post-operative analgesia. Animals were placed in a stereotaxic frame (Kopf instruments, United States) and MetaQuant microdialysis probes (PAN membrane, BrainLink, Netherlands) were implanted into the prefrontal cortex (PFC), 4 mm exposed surface. Coordinates for the tips of the probes for the PFC are: antero-posterior (AP) = +3.4 mm from bregma, lateral (L) = −0.8 mm from midline and ventral (V) = −5.0 mm from dura, the toothbar set at −3.3 mm. Microdialysis was performed approximately 24 h following the surgery. The microdialysis probes were connected with flexible PEEK tubing to a microperfusion pump (Harvard PHD 2000 Syringe pump, Holliston, MA). Microdialysis probes were perfused with artificial cerebrospinal fluid (aCSF) containing 147 mM NaCl, 3.0 mM KCl, 1.2 mM CaCl_2_, 1.2 mM MgCl_2_ + 0.15% BSA, at a slow flow rate of 0.15 μL/min and a carrier flow (ultra-pure +0.15% BSA) at a rate of 0.8 μL/min. Microdialysis samples were collected for 30 min periods by an automated fraction collector (820 Microsampler, Univentor, Malta) into 300 µL polypropylene mini-vials. After stabilization, one baseline sample was collected, the test compound (dexmedetomidine) was dissolved ultra-pure water at 5 µL or 50 µL and given sublingually. After that 8 additional 30-min interstitial fluid (ISF) samples from the PFC were collected for a total collection time of 5 h. In addition to ISF collection, blood samples were collected via the JV cannula at 0, 30, 60, 120, 180 and 240 min after dosing into EDTA anticoagulant vials (∼200 µL blood per sample) and kept on ice until processed for plasma by centrifugation at 2,500 *g*, 4°C for 10 min.

### 2.5 Forced swim test

#### 2.5.1 Site

Studies were conducted by Eurofins Advinus Ltd., Bengaluru, India. All procedures were followed as per the guidelines provided by the Committee for Purpose of Control and Supervision of Experiments on Animals (CPCSEA; since 2023 it is CCSEA) India. The study design and procedures followed herein have been approved by the Institutional Animal Ethics Committee (IAEC) of Eurofins Advinus Limited.

#### 2.5.2 Animals

A total of 78 male SD rats (9–10 weeks old) were procured from Hylasco Biotechnology Pvt. Ltd., Hyderabad, India and were divided into seven groups (9–12 animals per group). Animals were housed in an environmentally controlled room at 22°C ± 3°C and relative humidity of 30–70 percent. Adequate fresh air supply of 12–15 air changes/hour was maintained. The temperature was maintained at 20°C–23°C. Relative humidity was maintained at 58%–67% and the photoperiod was 12 h light and 12 h dark cycle. The lights of the acclimatization area were switched off at 06:00 p.m. Two rats per cage were housed in sterilized suspended standard, polysulfone cages, (Size: approximately L425 x B266 x H185 mm), with stainless steel top grills having facilities for holding pelleted food and drinking water in polycarbonate bottles with stainless steel sipper tubes. During the study period animals were housed in a single room. Animals received food and water *ad libitum*.

#### 2.5.3 Procedure

The test was carried out in transparent cylindrical glass containers measuring 46 cm in height and 20 cm in diameter. The containers were filled with water (23°C–25 °C) to a depth of 30 cm. Clean drying cages, heat lamps and heat pads were used for the animals that have finished the procedure to avoid hypothermia. The test was carried out in Dark Phase. There were 2 swim sessions, 24 h apart. The first session, Swim 1, was the pretest/training stage (15 min) and the second session, Swim 2, was the test stage (6 min). A red bulb of 50 W was used for observation during Swim 1 and Swim 2 procedure. Swim 1 was carried out between 18:30 to 21:30 and Swim 2 was carried out between 19:30 to 21:30.

On test day, animals were dosed intramuscularly with respective treatment 60 min prior to performing FST. Post injection, animals were observed for any abnormal effect due to intramuscular injection. In swim 2, the first 1-min data was excluded from analysis and the remaining 5 min data was considered.

Rats were placed in the water-filled cylinder for 6 min during Swim 2. Animals were observed for immobility. A rat was judged to be immobile when it remained floating in the water without struggling and was making only those movements necessary to keep its head above water. The duration of time spent immobile was observed by an observer blind to the treatment group. Water was changed after every session to avoid any influence on the next rat. Rats (9–12 animals per group) were administered vehicle (0.1 mL, intramuscular), desipramine hydrochloride (30 mg/kg, per oral) or dexmedetomidine (1 or 5 μg/kg, intramuscular), 1 h prior to test. Both desipramine and dexmedetomidine were formulated in normal saline. The rats were placed in a swim chamber and behaviors were measured for 5 min on test day. Data was presented as total immobility time (in seconds) over the 5-min trial.

### 2.6 Open space swim test

#### 2.6.1 Site

Study was conducted by Porsolt S.A.S., France. The study was conducted in compliance with Animal Health regulations, in particular: A) In accordance with the Porsolt facility accreditation for experimentation; and B) In accordance with the recommendations of the Association for Assessment and Accreditation of Laboratory Animal Care (AAALAC).

#### 2.6.2 Animals

54 male Swiss mice (18 animals per group), including 4 spare mice (6 weeks old, weighing 26–39 g at the beginning of testing on Day 1) were supplied for the study from Janvier Labs (53940 Le Genest-Saint-Isle, France). Mice were housed grouped in makrolon cages (no more than 6 animals per cage) on wood litter (SAFE, 89290 Augy, France). Environmental enrichment (such as tunnel, gnawing material, nesting material, etc.) were provided. The animal house was maintained under artificial lighting (12 h) between 7:00 and 19:00 in a controlled ambient temperature of 22°C ± 2°C, and relative humidity between 30% and 70%. All animals were provided with free access to food (Code A04 - SAFE, 89290 Augy, France) and water.

#### 2.6.3 Procedure

The open space swim test task is derived from that described by Porsolt et al. (1977). Mice that are placed in a cylinder of water from which they cannot escape rapidly turn immobile. Acute antidepressants decrease the duration of immobility in normal Swiss mice. Repeated exposure to the swimming task in a large open space bath increases immobility time (Sun and Alkon, 2003) in the rat and adapted to the mouse (Stone et al., 2008). Mice were individually placed in a makrolon cage (41 × 25 × 18 cm) containing 10 cm water (22°C) from which they could not escape, for 15 min daily during 5 consecutive days (Day 1 to Day 5) and then, on Days 8, 11, 15, 18, 22, 25, 26, 29 and 32. On days 8–32, test drug was administered as indicated in Figure 3. The observers were obscured from sight of the mice during the trials but were able to observe the animals’ behaviors on a video screen monitor during the trials. All swimming sessions were video-recorded, and the behavior of animals was analyzed using a video-tracking system (Panlab: SMART). The principal measures taken were the duration of immobility and the distance travelled during each trial. The time spent in the periphery of the arena was also measured. Data with the test substance was analyzed by comparing treated groups with vehicle control using two-way ANOVA followed by post-hoc Dunnett’s test.

### 2.7 CCK-4 elevated plus maze test

#### 2.7.1 Site

The study was conducted by Neurofit, France. The experiments were conducted in compliance with animal health regulation–in particular; A) with the legislation and regulations of French law (European Directive 2010/63/EU incorporated in French law, amended by Decree No. 2013–118); B) in compliance with Association for Assessment and Accreditation of Laboratory Animal Care International (AAALAC).

#### 2.7.2 Animals

48 male Wistar rats were supplied by Janvier (Le Genest St Isle, France) for this study. They were purchased at a body weight of 150 g and reached a body weight of about 270 g at the time of use. Animals were group-housed (3-4 rats per cage) and maintained in a room with controlled temperature (21°C–22°C) and a reversed light-dark cycle (12 h/12 h; lights on: 17:30–05:30; lights off: 05:30–17:30) with food and water available *ad libitum*.

#### 2.7.3 Procedure

Peripheral administration of CCK-4 leads to an anxiogenic-like action in the rats as assessed in the elevated plus maze (EPM) paradigm (Rex et al., 1994). In the present study, 12 animals per group were used. The apparatus is a PVC maze covered with Plexiglas and subdivided into four equal exploratory arms (40 × 10 cm), which are all interconnected by a small platform (10 × 10 cm). The apparatus is placed 65 cm above the floor. Two arms are open, and two others are closed with transparent wall (high: 10 cm).

After compound administration, a rat is placed on the platform opposite a closed arm. The number of entries and the time spent on each arm are recorded during a 5 min period. The animal is considered as entered in an arm when it places its four paws in the arm. The apparatus was cleaned between each animal using alcohol (70%). Urine and feces were removed from the maze. Data were analyzed by One way ANOVA followed by Dunnett’s comparisons test. CCK-4 (0.2 mg/kg) was administered intraperitoneally in the Wistar rats 30 min prior to the test. Dexmedetomidine (1, 2.5, 5 and 20 μg/kg) was administered i. p. 60 min prior to the study (30 min prior to CCK-4 administration). Diazepam (1 mg/kg) was administered 30 min prior to study (at the time of CCK-4 administration). After the administration of compounds, rats were placed on the platform opposite a closed arm in the elevated plus maze. The number of entries and the time spent in each arm were recorded during a 5 min period. The animal was considered as entered in an arm when it placed its four paws in the arm.

### 2.8 REM and slow wave sleep

#### 2.8.1 Site

Studies were conducted by PsychoGenics Inc. (Paramus NJ). All housing and testing of the animals were in accordance with the Principles of Laboratory Animal Care and the approval of the PsychoGenics, Inc. Institutional Animal Care and Use Committee in Association for Assessment and Accreditation of Laboratory Animal Care International (AAALAC).

#### 2.8.2 Animals

Twelve adult male SD rats (300–350 g) from Envigo (Indianapolis, IN) were housed 3 rats per cage and kept at 12/12 light/dark cycles. Chow and water were provided *ad libitum* for the duration of the study. Following surgery, rats were single housed for recovery (7–10 days) and for recording transferred to an electroencephalography (EEG) recording room.

#### 2.8.3 Procedure

Animals were anesthetized with 4%–5% isoflurane and 1 L/min oxygen for induction prior to surgery and maintained during surgery with 1%–2.5% isoflurane/1 L/min oxygen mix. Following preparation for surgery, a 3–4 cm incision was made on the midline on the top of the skull extending 1 cm posterior to the midpoint of the eyes to the base of the skull and extending to the mid dorsal neck region. A subcutaneous pocket was formed from the posterior end of the incision and the transmitter (DSI telemetry devices F50-EEE) was placed such that the lead wires were extending out of the neck incision. Lead wires attached to the transmitter were inserted in one of the dorsal neck muscles and secured in place by suture. Using skull landmarks, bregma and lambda stereotaxic coordinates were Frontal/Parietal (right): (+) 2 mm Anterior, 2 mm Lateral; (−) 4 mm Posterior, 2 mm Lateral. Frontal/Frontal (bihemisphere): (+) 2 mm Anterior, 2 mm Lateral (right); (−) 2 mm Anterior, 2 mm Lateral (left). For each co-ordinate, a hole was drilled through the skull ensuring exposure of dura. Dural contact screws were inserted, and the EEG leads connected. Screws and surrounding skull surface area were permanently sealed with dental cement. Following drying, the skin overlying the skull was closed using sutures. EEG signals were filtered for line noise, artifacts were removed, and signal strength was checked to be between 100–500 μV.

Recordings started 2 h prior to compound administration (by intramuscular injection) and recorded continuously for 24 h thereafter. Rats were tested twice weekly in a cross over design with a minimum of 3-day washout period between doses. EEG data were read into NeuroScore software (Data Sciences International) for visualization, processing, and analyses. Sleep stages were assigned in 10-s epochs using EEG, electromyography, and locomotor activity. Four sleep stages were defined: Active Wake; Quiet Wake; slow wave sleep (SWS), and paradoxical or REM sleep. Fast Fourier Transform was used for spectral analysis. Spectral power was measured for each 10-s epoch at 1 Hz resolution (1–100 Hz) and summed to yield cumulative power. Recordings started 2 h prior to dexmedetomidine (5 and 20 μg/kg) or vehicle administration (by intramuscular injection) and recorded continuously for 24 h thereafter. SD rats (N = 11–12 per group) were tested twice weekly in a cross over design with a minimum of 3-day washout period between doses. EEG and electromyography (EMG) data were read into NeuroScore™ Software (Data Sciences International) for visualization, processing, and analyses. Sleep stages were assigned in 10-s epochs using EEG, EMG, and locomotor activity. Four sleep stages were defined: Active Wake; Quiet Wake; slow wave sleep (SWS), and paradoxical or rapid eye movement (REM) sleep. Fast Fourier Transform was used for spectral analysis. Spectral power was measured for each 10-s epoch at 1 Hz resolution (1–100 Hz) and summed to yield cumulative power.

### 2.9 Cued fear conditioning test

#### 2.9.1 Site

The study was conducted by Suven Life Sciences Limited (Hyderabad, India). The study was conducted in compliance with the Institutional Animal Ethics Committee (IAEC) requirements and in-house animal care and usage policy.

#### 2.9.2 Animals

32 male Wistar rats (7–8 weeks old, 232–289 g) were used for fear conditioning test. Rats were housed 1 animal per cage in sterilized solid bottom polycarbonate cages (Dimensions 42.5 cm [L] X 26.5 cm [W] X 18.5 cm [H]) with stainless steel grill tops, facilitate for food and water bottle, and bedding of corncob husk. Standard laboratory conditions, temperature was maintained between 21°C–22 C and relative humidity between 50% and 53% under a 12 h light/dark cycle (lights on between (07:00–19:00 h). Pelleted rodent feed manufactured by SAFETM laboratory (France) diet source, was provided. Food and water were supplied *ad libitum*.

#### 2.9.3 Procedure

Rodent fear conditioning chambers (L x W x H, rat: 26 × 30 × 33 cm, Coulbourn instruments. United States) placed in sound attenuating cabinets were used. Stainless steel grid floors (L x W, rat: 26 × 30 cm), connected to shock scramblers were used to deliver the shock. A ventilation fan provided a background noise of 60 dB. A light bulb (centered on the right wall, and 27 cm above the floor of the chamber) provided ambient illumination. Experiments were recorded using video camera, mounted on the cabinet ceiling and images were analyzed with freeze frame 3 software (V3.2.1). The fear conditioning chambers have transparent acrylic panels. All experimentations were carried out in a single fear conditioning chamber.

On day 0, animals were randomized into eight groups based on their body weight.

Session-I: On day 1 (N = 5), rats were brought to the laboratory at least 1 h prior to experimentation. The fear conditioning chamber was maintained with transparent acrylic panels. Rats were acclimatized to fear conditioning chamber for 1 min. After acclimatization rats from groups 1 to 4 received conditioned stimulus (CS) (2 kHz tone at ∼85 dB was delivered for 10 s) and an unavoidable foot shock (unconditioned stimulus (US): electric shock of 0.7 mA for 3s). US coupled with the last 3 s of CS. Following a 40 s interval between each administration, shock was repeated to deliver a total of six CS-US pairings. 40 s after the last US, the animal was transferred to the home cage. Rats were treated with respective treatments (vehicle, dexmedetomidine or scopolamine) 30 min before the conditioning. The duration of freezing was recorded by the software for 0–360 s simultaneously during the paired conditioning (defined as no movement for about of 3 s or more was scored a freezing behavior). The freezing threshold was set at 10 in the motion index for analysis. The chamber was cleaned with 70% v/v ethanol between tests.

On day 2, rats were brought to the laboratory at least 1 h prior to experimentation. Rats were acclimatized to fear conditioning chamber for 1 min. After acclimatization rats from group number 1-4 received CS (2 kHz tone at ∼85 dB was delivered for 10 s). Following a 40 s interval between each administration, tone was repeated to deliver a total of six CS. The freezing behavior of the animals was recorded for 6 min (starting from the time the animal placed in the fear conditioning chamber). After the 6 min behavioral recording, the animal was transferred to the home cage. For each treatment group, duration of freezing was compared against the vehicle by using one-way ANOVA followed by Dunnett’s comparisons test.

### 2.10 Rotarod test

#### 2.10.1 Site

Study was conducted by Porsolt S.A.S., France. The study was conducted in compliance with Animal Health regulations, in particular: A) In accordance with the Porsolt facility accreditation for experimentation; and B) In accordance with the recommendations of the Association for Assessment and Accreditation of Laboratory Animal Care (AAALAC).

#### 2.10.2 Animals

75 male Swiss mice (6 weeks old, 29–41 g body weight range at the beginning of the experiment) were supplied by Janvier Labs (Le Genest St Isle, France). Mice were housed grouped in makrolon cages (no more than 4 animals per cage) on wood litter (SAFE, 89290 Augy, France) with free access to food (Code A04 - SAFE, 89290 Augy, France) and water. Environmental enrichment such as gnawing and nesting material was provided. The animal house was maintained under artificial lighting (12 h) between 7:00 and 19:00 in a controlled ambient temperature of 22°C ± 2°C.

#### 2.10.3 Procedure

On Day 0, mice were submitted to two training trials separated by a 5-min interval. For each trial, mice were placed on a rod (diameter: 7 cm) rotating at a speed of 18 revolutions per minute for a 3-min period. On Day 1, a final training was performed in the morning (at least 2 h before the drug testing). On Day 1, the test session was performed at least 2 h after the habituation period (in the afternoon) and mice were placed on the rod for a maximum period of 3 min. The latency to fall off the rod was recorded. Data was analyzed by using one-way ANOVA followed by Dunnett’s comparisons test.75 male Swiss mice (6 weeks old, 29–41 g) were allocated into five groups (10–15 animals per group). Dexmedetomidine (20, 40 and 80 μg/kg; 60 min prior to test session) and diazepam (4 mg/kg; 30 min prior to test session) were administered intraperitoneally as per groups allocation.

### 2.11 Data analysis

Sizes of the experimental groups for each of the *in vivo* studies reported here were determined from the validation of the assays by the respective CROs. The group size was based on a consistent significant response of the respective positive control in each of the tasks.

All the behavioral data were analyzed using GraphPad Prism^®^ software (Version 10.2.3). Data from FST, EPM (CCK-4), sleep, rotarod and fear conditioning studies, were analyzed using one-way ANOVA followed by Dunnett’s test. OSST data was analyzed by Welch’s unpaired t-test. In all analyses, *p* values <0.05 were considered significant. All the behavioral data were acquired by observers that were blind to the treatment group.

## 3 Results

### 3.1 *In Vitro* pharmacology

**FIGURE 1:**
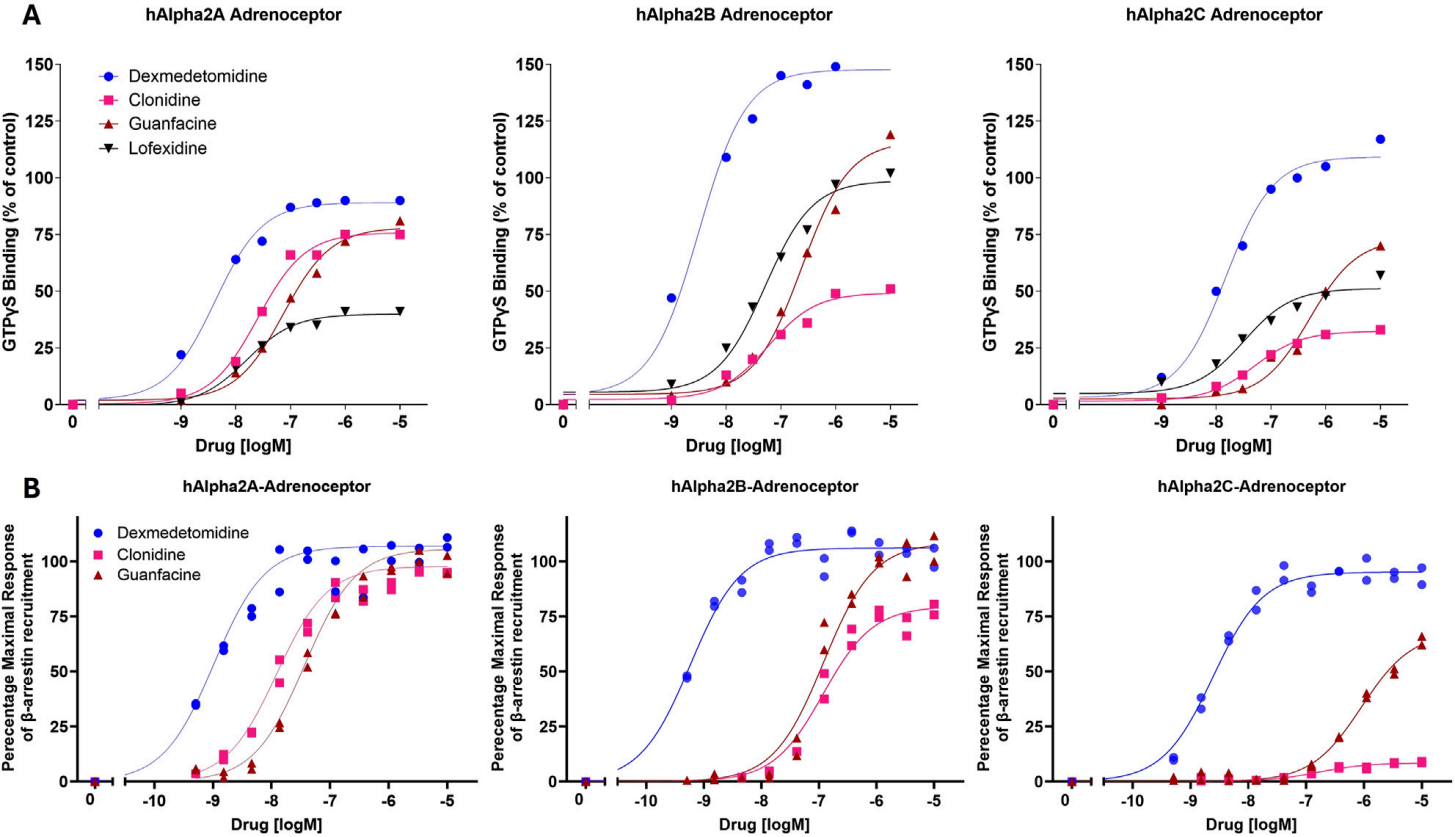
*In vitro* potency and intrinsic activity of alpha2-adrenoceptor agonists. **(A)** Alpha2-adrenoceptor-mediated stimulation of [^35^S]GTPγS binding to G proteins was measured in cell membranes expressing human α2_A_-, α2_B_- or α2_C_- adrenoceptors. Membranes were incubated with indicated concentrations of dexmedetomidine, clonidine, guanfacine or lofexidine. The data points were normalized using the maximal response achieved by the positive control, UK-14304 (brimonidine). Each point represents the mean of two replicates. **(B)** Alpha2 adrenoceptor-mediated recruitment of β-arrestin in cell membranes expressing human α2_A_-, α2_B_- or α2_C_- adrenoceptors.

Dexmedetomidine stimulates GTPγS binding to cells transfected with cDNA encoding human alpha-2 adrenergic receptors (α2_A_-, α2_B_- or α2_C_- adrenoceptors) (Figure 1A; Table 1). Dexmedetomidine demonstrated the same pattern of activity when β-arrestin recruitment was used as the endpoint (Figure 1B; Table 2). Dexmedetomidine is more potent and achieves a higher maximal response than approved drugs of the α_2A_ adrenergic class including clonidine, lofexidine or guanfacine. Our results are qualitatively similar to those reported previously (Jasper et al., 1998; Audinot et al., 2002; Fink et al., 2022). Of note is that lofexidine is only weakly active at α_2A_ adrenergic receptors, less than half the activity of dexmedetomidine. Also, clonidine appears to exhibit very low intrinsic activity at the α_2C_-AR and would presumably act as an antagonist at that receptor. The fact that these results with clonidine repeated in two different assay systems, with different membrane batches, supports this as real difference between clonidine and dexmedetomidine and not an outlier. Finally, as initially reported by Fink et al. (2022), dexmedetomidine appears to be nearly equally potent at both the GTP-binding and β-arrestin pathways and does not appear to be a biased ligand, meaning it does not preferentially activate either the GTP-binding or β -arrestin pathway.

**TABLE 1:** Agonist of alpha_2_-adrenergic receptor potency and efficacy in stimulating [^35^S]GTPγS binding to membranes expressing human α_2A,_ α_2B_ or α_2C_ receptors. Concentration–response curves were analyzed by nonlinear regression using a 3-parameter logistic equation. Agonist potency is expressed as the concentration required for half-maximal activation relative to the E_max_ of each compound. Maximal activity is expressed as the maximal response obtained by each compound relative to the maximal response achieved by the reference agonist, UK-14304.

Drug	α_2A_R		α_2B_R		α_2C_R	
	EC_50_ (nM)	Max activity (%)	EC_50_ (nM)	Max activity (%)	EC_50_ (nM)	Max activity (%)
Dexmedetomidine	3.9	89	2.8	147	14	110
Clonidine	25	76	49	49	43	32
Guanfacine	69	78	2010	110	45	72
Lofexidine	17	40	43	97	23	50

**TABLE 2:** Agonist of alpha_2_-adrenergic receptor potency and efficacy in stimulating β-arrestin binding to membranes expressing human α_2A,_ α_2B_ or α_2C_ receptors. Concentration–response curves were analyzed by nonlinear regression using a 3-parameter logistic equation. Agonist potency is expressed as the concentration required for half-maximal activation relative to the E_max_ of each compound. Maximal activity is expressed as the maximal response obtained by each compound relative to the maximal response achieved by the reference agonist, UK-14304.

Drug	α_2A_R		α_2B_R		α_2C_R	
	EC_50_ (nM)	Max activity (%)	EC_50_ (nM)	Max activity (%)	EC_50_ (nM)	Max activity (%)
Dexmedetomidine	1	101	0.6	106	24	95
Clonidine	13	93	118	80	160	8
Guanfacine	38	100	120	109	910	68

### 3.2 Dexmedetomidine microdialysis

**FIGURE 2:**
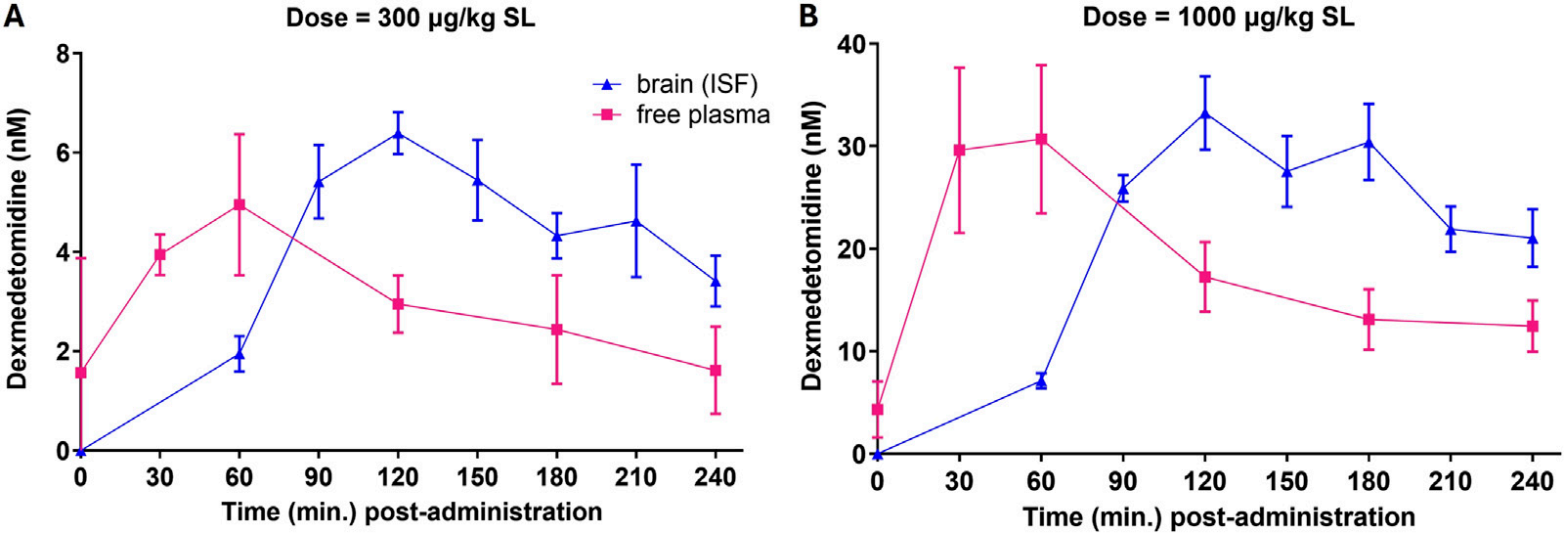
Free plasma concentrations and free brain concentrations after dosing. Rats (n = 5) were dosed sublingually (SL) at 300 μg/kg **(A)** or 1,000 μg/kg **(B).** Plasma samples and brain levels (interstitial fluid from microdialysis probes) were obtained at the indicated time points. Values are means ± SEM. Dexmedetomidine concentrations (ng/mL) were converted to nanomolar for comparison to *in vitro* potency.

Free brain concentrations of dexmedetomidine were measured using microdialysis probes after sublingual dosing. Free brain concentrations were compared to the free plasma concentrations assuming that dexmedetomidine is 84% bound to plasma proteins (Bol et al., 1997). Plasma levels reached a maximum value between 30 and 60 min and free brain at 90–120 min (Figure 2). Free brain levels were still approximately the same as peak plasma levels at 6 h, the last time point measured. These findings were similar with both the 300 and 1,000 μg/kg dose. Extrapolating these free brain concentrations to lower doses used in the behavioral studies allows estimation of the level of occupancy required to achieve a behavioral response. For the 1,000 μg/kg dose, the peak free brain concentration was 30 nM. The predicted free brain concentration for a 10 μg/kg dose would be 0.3 nM, close to the predicted EC_50_ in the β -arrestin assay, 1 nM (Table 2).

### 3.3 Forced swim test

**FIGURE 3:**
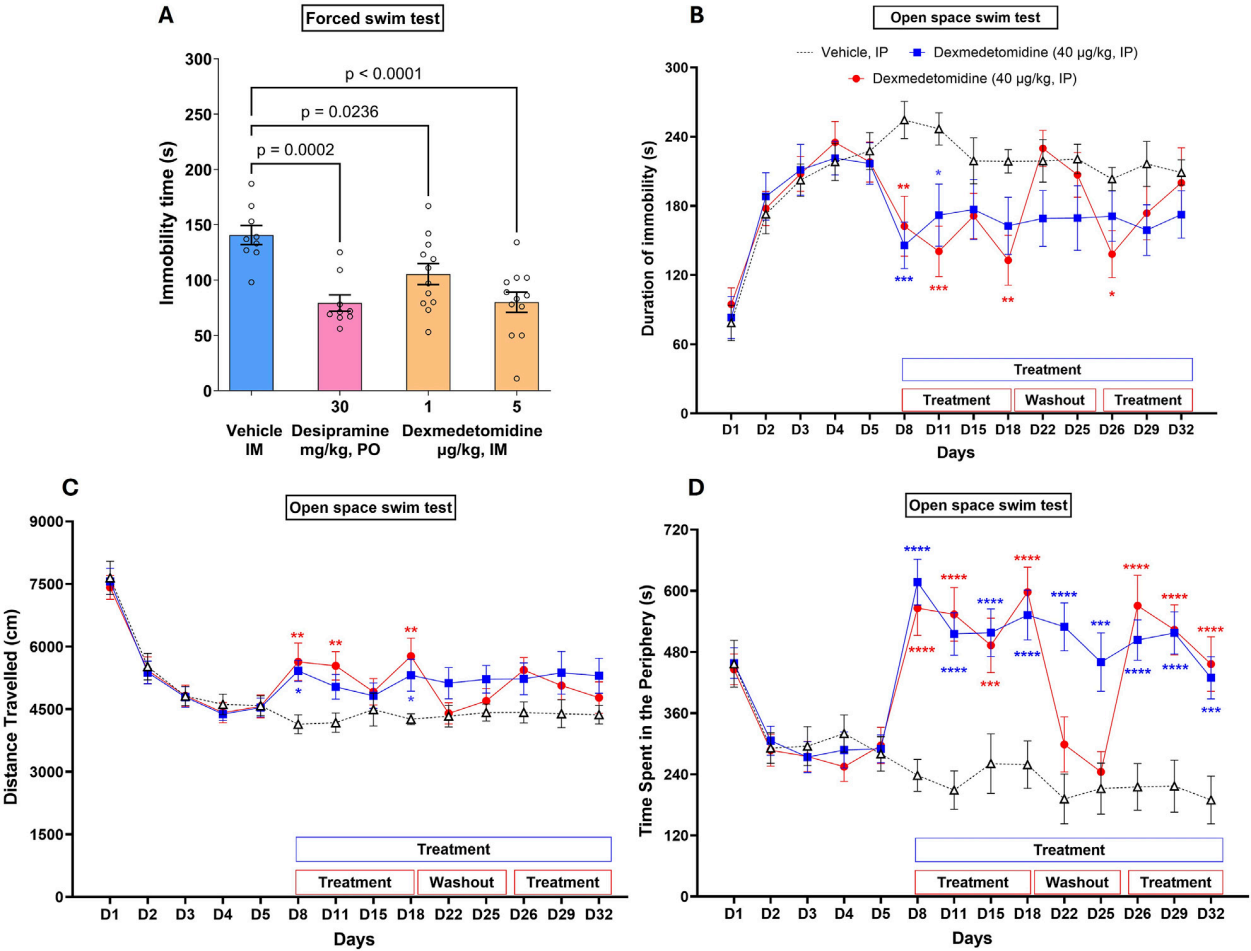
Acute dose **(A)** and multiple repeated dose **(B–D)** effects of dexmedetomidine on stress-induced immobility. **(A)** Rats (n = 9–12 per group) were dosed acutely with vehicle, dexmedetomidine at one and 5 μg/kg (intramuscular) or the positive control desipramine and then placed into a cylinder of water. Immobility time was recorded. Data were analyzed using one-way ANOVA followed by Dunnett’s multiple comparisons test and represented as mean ± SEM. **(B–D)** Mice (n = 18) were exposed to repeated stress for 5 days to increase immobility time. After day 5, mice were dosed on the days indicated in the figure. Dexmedetomidine (40 μg/kg, intraperitoneal) decreased immobility time **(B)**, increased distance travelled **(C)** and increased time spent in the periphery **(D)** on treatment days as indicated. Data was analyzed using two-way ANOVA followed by Tukey’s multiple comparisons test and represented as mean ± SEM. Statistical comparison of repeated daily treatment of dexmedetomidine (from D8 to D32) with corresponding vehicle control is depicted with # symbol and that of repeated daily treatment of dexmedetomidine (from D8 to D18 and from D26 to D32; with vehicle treatment from D19 to D25) with corresponding vehicle control is depicted with * symbol.

The forced swim test, also called the behavioral despair test, is a behavioral task in which animals are acutely stressed by being placed in a cylinder filled with water after which they adopt an immobile behavior. Dexmedtomidine at 1 and 5 μg/kg significantly decreased immobility time (p = 0.0238 and p < 0.0001, respectively, versus vehicle group) indicating a positive adaptation to stress (Figure 3A). Dexmedetomidine data was qualitatively similar to the effect of the positive control, 30 mg/kg of the antidepressant imipramine (p = 0.0002 versus vehicle). Data were analyzed using one-way ANOVA (F_3,38_ = 9.4, p < 0.001) followed by Dunnett’s multiple comparisons test and represented as mean ± SEM (n = 9–12 per group).

### 3.4 Open space swim test

Open space swimming test (OSST) is qualitatively similar to the forced swim test except that the rodents are repeatedly exposed to stress and investigational drug is administered repeatedly over 25 days.

### 3.5 Swimming behavior before initiation of treatment (Days 1–5)

After 3 days of being placed into the water-filled cylinder, immobility greatly increased and distance travelled and time spent at the periphery of the cylinder decreased (Figures 3B–D). The effect of day of stress on immobility was significant (F _4,255_ = 35.7; p < 0.0001). Immobility time remained stable from Day 3 to Day 5. No significant differences were observed between the three groups of mice before starting drug treatment.

#### 3.5.1 Evaluation of acute treatment (Day 8)

Dexmedetomidine, on days 8–18, caused a significant decrease in immobility time (Figure 3B; F2,204 = 17.8, p < 0.0001). Dexmedetomidine (40 μg/kg), acutely administered, i. p., 60 min before the swimming session on Day 8, significantly decreased the mean duration of immobility (Figure 3B) and significantly increased the mean distance travelled (Figure 3C) and time at the periphery (Figure 3D). On day 8, the first day of dosing, dexmedetomidine decreased immobility time (p = 0.001 and p = 0.0067, respectively, for the two dosed groups versus vehicle). The mean distance travelled increased (+31%, *p* < 0.05 and +36%, *p* < 0.01), and the mean duration of immobility decreased (−43% and −36%, *p* < 0.01), respectively, for both treated groups as compared with vehicle controls. Dexmedetomidine also significantly increased the time spent in the periphery (Figure 3D, +159% and +138%, *p* < 0.001 for both treated groups, respectively). Data were analyzed using two-way ANOVA followed by Tukey’s multiple comparisons test and represented as Mean ± SEM (N = 18 per group). Each comparison of treated mice was made versus vehicle treated mice on the same day.

### 3.6 Evaluation of the effects of treatment after repeated administration (Days 8–32)

In vehicle controls, no clear variations were observed in the mean distance travelled, the mean duration of immobility or the time spent in the periphery from Day 8 to Day 32. Dexmedetomidine, administered daily, i. p., from Day 8 to Day 32 (60 min before the swimming session on testing days), decreased the mean duration of immobility over the testing period up to Day 32 (F_2,459_ = 19.9; p < 0.0001). It also increased the mean distance travelled (F_2, 459_ = 19.4; p < 0.0001), and increased the time spent in the periphery (F_2,459_ = 99, p < 0.0001) over the testing period.

### 3.7 Effect of treatment during and after washout period

When the dexmedetomidine treatment was stopped from Day 19 to Day 25 (group 3), treated animals displayed similar swimming behavior compared to vehicle controls on testing Days 22 and 25.

### 3.8 Rotarod test

Following the last training session, 15 out of 75 mice failed to stay on the rotarod for the whole 3-min period, and were discarded for the drug test session, which resulted in N = 10–13 per group for the drug test session. During the drug test session, vehicle controls displayed a mean latency to fall-off 154.8 ± 14.7 s.

**FIGURE 4:**
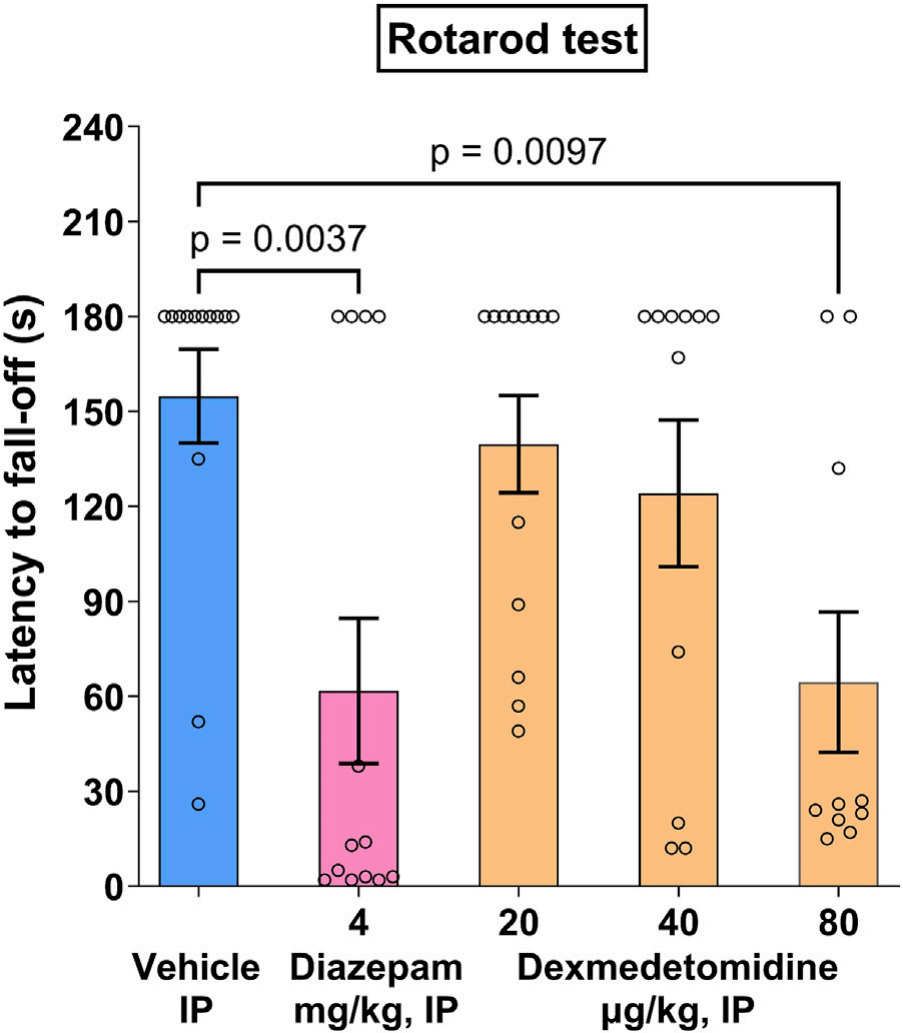
Rotarod effects in mice. Dexmedetomidine, at doses of 20, 40 and 80 μg/kg, was assessed on latency to fall-off in the rotarod motor test in Swiss mice (n = 10–13). Data represents mean ± SEM.

Dexmedetomidine, at behaviorally relevant doses (20 and 40 μg/kg), did not affect mean latency to fall off (Figure 4). At higher doses, dexmedetomidine (80 μg/kg), administered i. p. 60 min before the drug test session, significantly decreased the latency to fall-off (−58%, *p* < 0.01) as compared with vehicle controls (Figure 4) (F _4, 55_ = 4.883). Diazepam (4 mg/kg), administered i. p. 30 min before the test, significantly decreased the latency to fall-off (−60%, *p* < 0.01) as compared with vehicle controls. Data was analyzed using one-way ANOVA followed by Dunnett’s test.

### 3.9 CCK-4 elevated plus maze test

**FIGURE 5:**
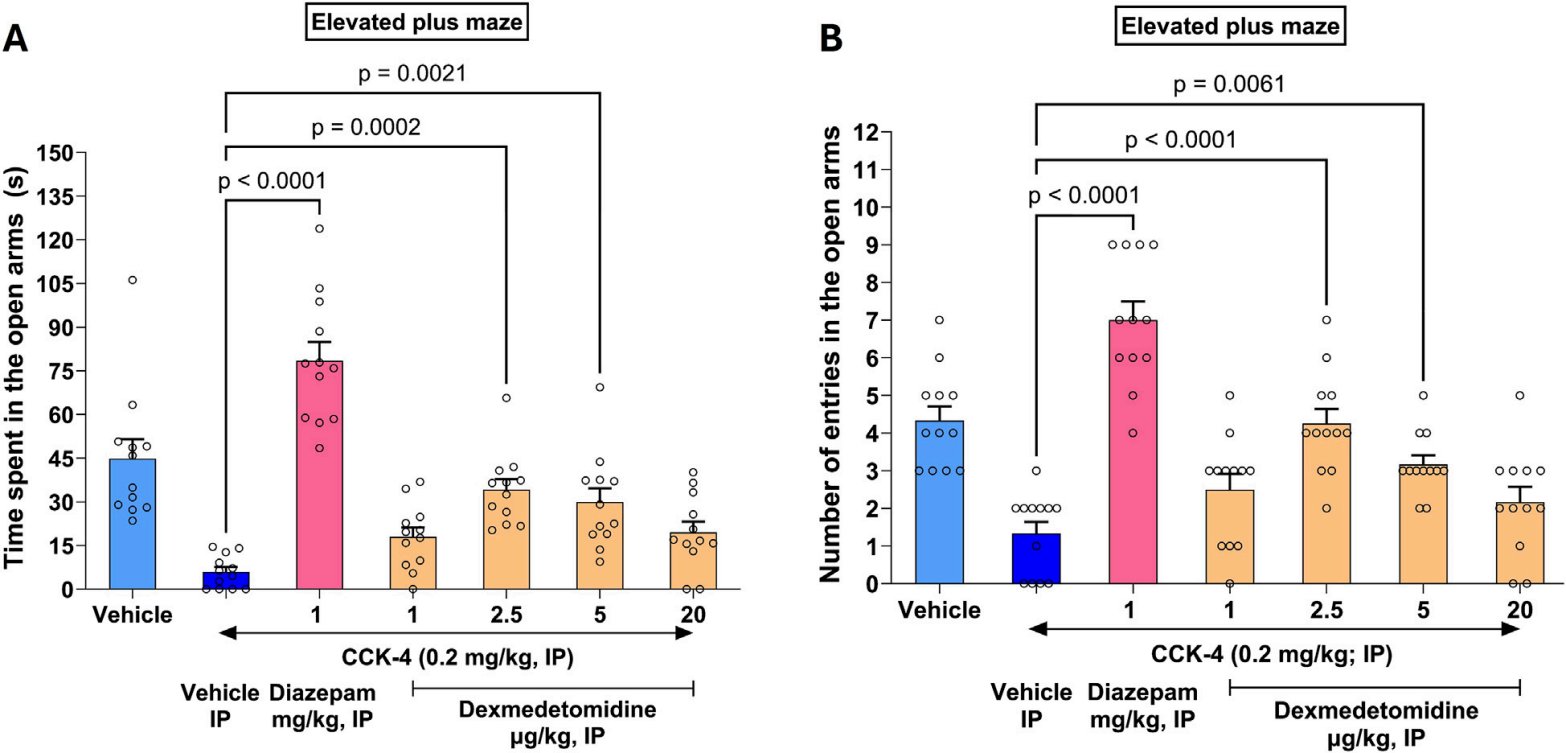
CCK-4 induced deficits in the elevated plus maze in Wistar rats. The saline vehicle (light blue bar on left) shows time spent **(A)** or entries **(B)** by rats in open arms of an elevated plus maze (EPM). Effects of CCK-4 on EPM is shown in the dark blue bar (Vehicle IP) to the right. Effects of the positive control diazepam (magenta) and increasing doses of dexmedetomidine are shown in combination with CCK-4. As expected, at the high sedating dose of 20 μg/kg, dexmedetomidine was not effective.

In rats, CC-4 elicits a state of anxiety as evidenced by a reluctance for the rats to explore the open arms of an elevated plus maze (EPM). CCK-4 reduced time spent in the open arms from 45 s to 6 s (Figure 5A). Similarly, CCK-4 reduced the number of entries in the open arms of the maze from 4.3 to 1.3 (Figure 5B). Effects of treatment significantly increased time spent in open arms compared to CCK-4 alone (F_6, 77_ = 26.9, p < 0.0001). Similarly, treatment affected entries into open arms versus CCK-4 alone (F_6, 77_ = 23.9, p < 0.0001). Effects of diazepam exceeded those measured in vehicle animals, suggesting that diazepam might have independent effects on the elevated plus maze not dependent on CCK-4.

Dexmedetomidine treatment reversed the deficit induced by CCK-4 nearly completely at doses of 2.5 and 5 μg/kg. The effect of dexmedetomidine was significant at doses 2.5 μg/kg (4.3 ± 0.4, 97%, *p* < 0.0001 and 34.2 ± 3.6 s, 73%, *p* = 0.0002, respectively) and 5 μg/kg (3.2 ± 0.2, 61%, *p* = 0.0061 and 29.9 ± 4.7 s, 62%, *p* = 0.0021, respectively). At a sedative dose, 20 μg/kg, time in open arms and entries into open arms returned to CCK-4 levels as expected. Data were analyzed using one-way ANOVA followed by Dunnett’s multiple comparisons test and represented as mean ± SEM (n = 12 per group).

### 3.10 REM and SWS

**FIGURE 6:**
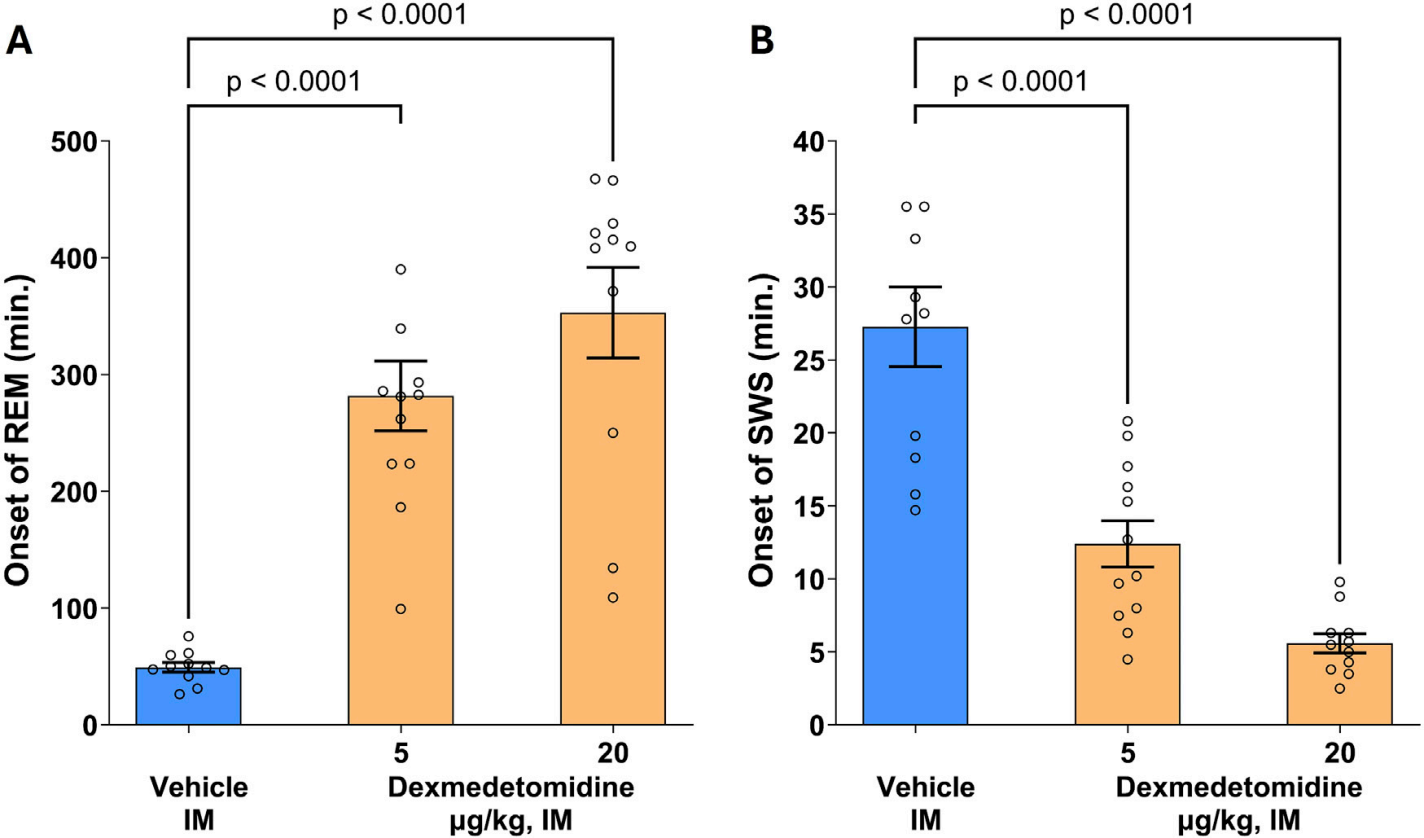
Onset of rapid eye movement (REM) and slow wave sleep (SWS). Male SD rats (n = 11–12 per group) were assessed using polysomnography to determine onset of REM and SWS. Dexmedetomidine (5 and 20 μg/kg, intramuscular) increased the latency to REM sleep **(A)** and decreased latency to onset of SWS **(B)**. Dexmedetomidine PSG recordings were scored for Wake, Active Wake, REM and SWS following dexmedetomidine or vehicle and the time post-dose to the first observable epoch (10s) classified as REM or SWS was measured. Data are means ± SEM.

Rat polysomnography (PSG) recordings were scored for four different sleep stages: active awake, awake, rapid eye movement (REM) and slow wave sleep (SWS). Dexmedetomidine had a robust, concentration-dependent effect on the post-dose onset time (latency) of REM sleep (Figure 6; F _2,31_ = 30.3, p < 0.0001). The onset of SWS was significantly reduced by both the 5 and 20 μg/kg doses (F _2,31_ = 35, p < 0.0001). Conversely, onset to REM sleep was significantly increased by approximately six to 8-fold. This profile indicates that dexmedetomidine suppresses REM sleep and increases SWS. Hypnogram data were analyzed using one-way ANOVA followed by Dunnett’s test.

### 3.11 Cued fear conditioning test

**FIGURE 7:**
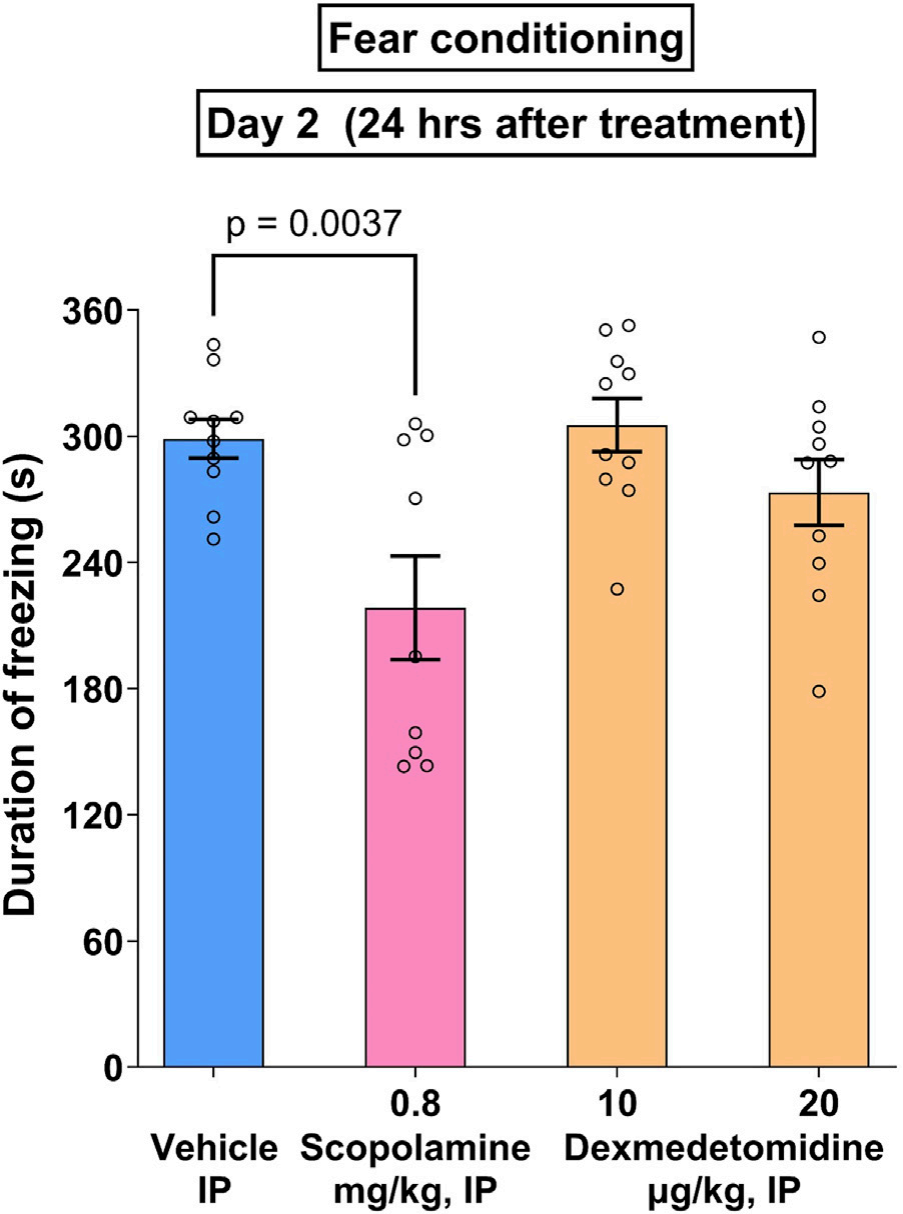
Cued fear conditioning. Male Wistar rats (n = 10 per group) were given paired associations of an unconditioned (shock) and conditioned (tone) stimuli as described in methods. The positive control, scopolamine (0.8 mg/kg), caused a significant reduction in freezing time that was not matched by dexmedetomidine (10 and 20 μg/kg). Data represents mean ± SEM of duration of freezing.

Noradrenergic signaling may either disrupt or enhance memory formation (Giustino and Maren, 2018). In the case of cued fear conditioning, an innocuous or conditioned stimulus (CS) such as a tone is presented to test animals at the same time as a noxious or unconditioned stimulus (US) such as a shock. If the animal remembers the association of the US with the CS, the animal will freeze when presented with the CS alone. Test compounds, scopolamine and dexmedetomidine, were administered prior to the CS-US pairing. In the study shown in Figure 7, an overall treatment effect was measured (F_3,35_ = 3.8; p = 0.0025). Within the treatment groups, only the positive control, scopolamine, exhibited a significant effect (p = 0.0037). Data were analyzed using one-way ANOVA followed by Dunnett’s test.

## 4 Discussion

Stress-related neuropsychiatric symptoms, including anxiety and agitation, are a major healthcare problem. Discovering new mechanisms that underlie stress-induced symptoms is essential for developing drugs that work quickly and effectively without the problems associated with antipsychotics and benzodiazepines (Lopez and Chen, 2024). One mechanism that contributes to stress is the LC-noradrenergic signaling pathway (Yamamoto et al., 2014). Stress activates the LC and alters the firing rate of LC neurons resulting in anxiety-related behaviors in mice (McCall et al., 2015). The LC in turn activates other stress pathways including the hypothalamus–pituitary–adrenal (HPA) axis (Morris et al., 2020). Excessive norepinephrine signaling contributes to symptomology associated with psychiatric disorders including schizophrenia (Mäki-Marttunen et al., 2020), bipolar disorder (Manji et al., 2003), post-traumatic stress disorder (Sherin and Nemeroff, 2011; Belkin and Schwartz, 2015) behavioral and psychological symptoms of dementia (BPSD) (Wang et al., 2009) and withdrawal associated with opioid use disorder (Delfs et al., 2000). Noradrenergic crises may also occur for other reasons including drug use (cocaine intoxication), genetic diseases (familial dysautonomia), and even viral infections including COVID-19 (Dillon et al., 2017; Konig et al., 2020; Richards and Le, 2025). A drug with a mechanism of action that safely treats noradrenergic crises, and related symptoms, would be of great potential value to clinicians and their patients.

Genetics, tissue expression patterns and pharmacology all suggest that the alpha_2_ adrenoceptors are key regulators of the stress response. The α_2_-AR is a critical element in the adaptation to stress because it modulates LC-mediated noradrenergic signaling (Benarroch, 2018; Hein et al., 1999). Mouse genetics indicate that ADRA2A knockout mice exhibit a higher level of anxious behaviors when stressed (Schramm et al., 2001). Mice with inactivated ADRA2C exhibited a heightened aggression response and response to a startle stimulus (Sallinen et al., 1998). Alterations affecting α_2C_-ARs expression have been linked to changes in behavioral responses to stress, including increased immobility in the forced swimming test and elevated corticosterone levels after repeated stress, highlighting their involvement in chronic stress scenarios. (Sallinen, et al., 1999). Gene polymorphisms in ADRA2B loci in human genomes result in phenotypes that are associated with impulsivity and poor responses to stress (Wirz et al., 2017; Xie et al., 2018). Tissue expression patterns show that α_2_-ARs are expressed in key brain regions related to stress. The α_2_-ARs are expressed on key brain regions that respond to stress, including LC (Schwarz and Luo, 2015) and bed nucleus stria terminalis (Shields et al., 2009; Fetterly et al., 2019). The α_2A_-ARs and α_2C_-ARs are upregulated in stress-related brain regions in tree shrews after chronic stress (Flügge et al., 2003). Pharmacologically, activation of α2_A_-ARs with agonists like guanfacine increase c-fos expression in brain areas related to stress (Savchenko and Boughter, 2011). In rats, α_2_-AR agonists, like clonidine, reduce stress-related behaviors (Tornatzky and Miczek, 1994) and, conversely, the antagonist yohimbine promotes anxiety-related behaviors in humans (Charney et al., 1984). Results in this study indicate that dexmedetomidine affects both acute (forced swim test) and chronic stress (open space swim test), at doses consistent with brain levels that would activate α_2_-ARs (Figure 3).

Dexmedetomidine possesses high potency and intrinsic activity at all 3 α_2_-ARs (Figure 1). Potency and activity were measured independently using either radioisotope-based GTPγS binding for activation of G-proteins and chemiluminescent-based signals for β-arrestin recruitment. These two pathways (G-protein activation and β-arrestin recruitment) may mediate different signaling events (Calebiro and Grimes, 2020; Fink et al., 2022). While G-protein signaling often mediates rapid and transient responses, β-arrestin pathways can initiate longer-lasting signaling events and modulate receptor desensitization and internalization. These distinct mechanisms are known to differentially influence physiological processes, including stress regulation. Interestingly, both assays indicate that clonidine and guanfacine are much less effective at the α_2C_-AR whether measured independently by either GTPγS binding or β-arrestin recruitment. The consequence is that dexmedetomidine would be more effective at suppressing LC activity through both α_2A_-ARs and α_2C_-ARs than either clonidine or guanfacine by affecting both low and high frequency LC activity.

Unbound (free) brain levels of dexmedetomidine were calculated using microdialysis probes. Measuring unbound levels of CNS drugs after dosing is an important characteristic of useful CNS drugs (Loryan et al., 2022). Sublingual dosing was used to match clinical dosing and to minimize first pass metabolism. By comparing the area under the curve (AUC) after sublingual dosing to the AUC in brain as measured by microdialysis, the brain bioavailability may be determined (Figure 2). For the 1,000 μg/kg dose, the AUC for the plasma compartment is 0.092 mg*h/L. For free brain, it is 0.016 mg*h/L. The ratio of free brain to total plasma is 0.017. Given that the free (unbound) plasma fraction is 16%, this indicates that the free drug is freely permeable. The consequence of this is that CNS free brain levels may be predicted by knowing the plasma concentration.

An effective dose of 10 μg/kg should achieve a maximal brain level of 0.3 nM if we extrapolate from brain levels achieved by the 1,000 μg/kg dose (Figure 2). Assuming an affinity of roughly 1 nM for the α_2A_-AR, brain levels of 0.3 nM would yield occupancy levels of 20%–25%. Efficacy at low occupancy is a desirable characteristic of a drug because it means a large portion of excess (spare) receptors convert in sensitivity of effects to variations in receptor number. In support of this hypothesis, clonidine loses much of its effectiveness after α_2_-ARs are inactivated whereas dexmedetomidine retains efficacy (Takano and Yaksh, 1991; Hayashi et al., 1995).

Dexmedetomidine was effective in both the forced swim test and the open space swim test tasks. These are related tasks, likely mediated by the LC (Stone and Lin, 2011; Nam and Kerman, 2016). Although the forced swim test model is often used as a screen for potential antidepressant activity, it is more appropriately a screen for stress-related behaviors (Commons et al., 2017). Dexmedetomidine reduced immobility time, comparable to the positive control desipramine, indicating it improved the animal’s adaptive strategy to the stress incurred during the forced swim test. The open space swim test is like the forced swim test except that the animals are tested repeatedly in the swim tank during a period of 32 days. Dexmedetomidine continued to be effective after repeated dosing indicating no tachyphylaxis to the anti-stress effect. When dosing was stopped, behavior reverted to vehicle control levels, indicating that effects of dexmedetomidine are reversible. Reversibility and lack of tachyphylaxis after repeat dosing are desirable qualities for any successful drug. Moreover, there was no evidence of impaired motor activity in the rotarod test using Swiss mice at doses that were efficacious in the open space swim task (40 μg/kg). The findings that low doses of dexmedetomidine are effective in the open space swim test suggests that dexmedetomidine at low doses is not sedating, but in fact was able to increase activity in the mice (reduce immobility). At the higher dose, 80 μg/kg, which achieves plasma concentrations likely to be like the plasma concentrations used to sedate patients in the clinic before surgical procedures, an effect on the rotarod was observed. This may be an unappreciated feature of dexmedtomidine, specifically that at low doses and plasma exposures (and by extension low brain levels), dexmedetomidine has an anti-agitation or anxiolytic effect but is not overtly sedating.

The CCK-4 induced panic response is a translatable biomarker for stress effects (Zwanzger et al., 2001). CCK-4 (Cholecystokinin tetrapeptide) has been reported to trigger panic attacks in healthy volunteers (Eser et al., 2009) and in patients with panic disorder (Bradwejn et al., 1991). Dexmedetomidine was effective in reducing CCK-4 induced anxiety as measured in the elevated plus maze, further establishing its potential as an anti-stress agent. Interestingly, the positive control in the study, the benzodiazepine diazepam, increases activity to a greater extent than vehicle alone (Figure 5), whereas dexmedetomidine at 2.5 and 5 μg/kg reverses the CCK-4 effect back to the vehicle level. This suggests that diazepam may be working independently of the CCK-4 deficit but dexmedetomidine does not.

Latency to REM sleep is a translatable biomarker for drugs with antidepressant effects and almost all antidepressants suppress REM sleep (Riemann et al., 2020). In the rats, dexmedetomidine both increased latency to REM sleep and shortened the latency to SWS. The effects of dexmedetomidine on sleep have been well documented in humans (Akeju et al., 2018; Ramaswamy et al., 2021). The effect on SWS is consistent with other reports that indicate dexmedetomidine increases SWS. It is during slow wave sleep that activation of the glymphatic system is thought to occur (Nedergaard and Goldman, 2020). The glymphatic system clears waste products from the brain and therefore increasing this clearance could have beneficial effects on brain function. Dexmedetomidine has recently been proposed as a potential glymphatic enhancer (Persson et al., 2022).

Norepinephrine signaling may be important to essential biological phenomena such as cognition, therefore, it is important to demonstrate that dexmedetomidine does not impair cognition at doses that are effective in stress-mediated tasks. In the cued fear conditioning task, an LC-dependent behavior (Giustino and Maren, 2018), dexmedetomidine did not impair memory formation at efficacious doses (Figure 7). Scopolamine, a well characterized amnestic drug that was used as a positive control in this task, behaved as expected. Therefore, dexmedetomidine does not produce a memory deficit under conditions in which scopolamine is amnestic. In humans, other α_2_-AR agonists like guanfacine appear to improve cognition in stress-related cognitive dysfunction (Arnsten et al., 2023). Intravenous dexmedetomidine, when used to sedate patients before surgical procedures, causes much less post-operative cognitive dysfunction than other sedating agents, like propofol (Karageorgos et al., 2025). This would be consistent with the idea that dexmedetomidine prevents stress-related cognitive dysfunction.

Limitations of this study include the need to examine the effects of dexmedetomidine on sleep, cognition and motor coordination under chronic conditions. Secondly, the translational value of latency to REM sleep and the CCK-4 panic model need to be established under chronic dosing conditions.

In conclusion, dexmedetomidine possesses many characteristics that make it an excellent drug for the acute treatment of stress-related symptoms resulting from hyper-noradrenergic signaling or hyper-arousal. In this study, the effects of dexmedetomidine were reversible and no loss in efficacy (tachyphylaxis or tolerance) was observed after repeat dosing. Effects occurred at low receptor occupancy meaning that efficacy would be less sensitive to changes in receptor number. No overt adverse effect on memory formation was observed at efficacious doses. Dexmedetomidine, unlike clonidine, lofexidine and guanfacine, appears to be a full agonist at all 3 α_2_-ARs, all of which have been associated with modulating responses to stress. Dexmedetomidine may be a useful drug to chronically treat a wide number of disorders that are mediated by norepinephrine signaling.

## Data Availability

The raw data supporting the conclusions of this article will be made available by the authors, without undue reservation.
